# Time to choose: impact of intertrial interval on selecting between methamphetamine and food reinforcement in male and female rats

**DOI:** 10.1007/s00213-025-06750-w

**Published:** 2025-02-08

**Authors:** Marlaina R. Stocco, Mari Purpura, Philip A. Vieira, Kira Wallquist, Sijia Wang, Julia Adams, Karen K. Szumlinski, Tod E. Kippin

**Affiliations:** 1 Department of Psychological and Brain Sciences, University of California, Santa Barbara, CA USA; 2 Department of Psychology, California State University, Dominguez Hills, Carson, CA USA; 3 Department of Psychiatry and Behavioral Sciences, University of California, San Francisco, San Francisco, CA USA; 4 Department of Molecular Cellular and Developmental Biology, University of California, Santa Barbara, Santa Barbara, CA USA; 5 Neuroscience Research Institute, University of California, Santa Barbara, Santa Barbara, CA USA

**Keywords:** Methamphetamine, Choice, Self-administration, Reinforcement schedule, Breakpoint, Cocaine, Dose, Sex difference, Intertrial interval, Addiction

## Abstract

**Rationale:**

A central component of substance use disorder is the maladaptive choice of the drug over natural reinforcers. Compared to other drugs of abuse, methamphetamine (METH) choice has received limited study.

**Objective:**

We sought to characterize the role of intertrial interval on METH choice behavior.

**Methods:**

We examined the choice of METH versus food, across multiple METH doses (0.05–0.2 mg/kg/infusion), between male and female rats, employing a fixed ratio (FR1) reinforcement schedule with intertrial intervals (ITIs) of 20 and 600 s. Rats learned to lever-press for either the METH or the food reinforcer during separate, alternating training sessions. Rats then underwent choice testing, where both levers were presented for 25 discreet trials per session. Lastly, under a progressive ratio (PR) schedule, breakpoints for METH and food were assessed during separate, alternating sessions.

**Results:**

METH choice was substantially higher when using the 20 s versus 600 s ITI. When the 20 s ITI was used, choice was dose- but not sex-dependent. When using the 600 s ITI, choice was influenced by dose and sex, with female rats in the higher dose group choosing METH more than other groups. PR breakpoints were higher for METH than for food, and this effect was more pronounced among female rats. METH choice was positively correlated with the ratio of METH/food breakpoints.

**Conclusion:**

Reinforcement schedule parameters, namely ITI, during discrete choice testing can markedly influence METH choice behavior; thus, this should be carefully considered during experiment design and selected based on overarching study aims.

**Supplementary Information:**

The online version contains supplementary material available at 10.1007/s00213-025-06750-w.

## Introduction

Drug abuse and substance use disorder are serious health issues in the United States and worldwide, and stimulants, such as amphetamines, are among the most widely used drugs of abuse (SAMHSA, 2019; UNODC, 2019). One of the core behavioral symptoms of stimulant use disorder is the neglect of alternative interests and other normally rewarding activities in favor of continued drug use (Lamb et al. 2016; Banks and Negus 2017; American Psychiatric Association 2022); i.e., important social, occupational, or recreational activities are forfeited in favor of increasingly compulsive drug acquisition and intake (American Psychiatric Association 2022). Among those who develop METH addiction, such a preference for, and choice of, drug over competing sources of reinforcement represents a critical component of abuse liability and substance use disorder pathology.

Animal models of drug choice, including METH choice, assess drug taking at the expense of other reinforcers, typically during a series of discrete trials where the drug and another reinforcer are available. These choice models importantly capture an ethological element of human drug taking by assessing behavioral allocation within a defined, albeit limited, economy where multiple outcomes can occur (Lamb et al. 2016; Banks and Negus 2017; Venniro and Shaham 2020; Venniro et al. 2022). Identifying subject-specific factors that determine METH choice in these paradigms should provide insight into the internal and external factors, which contribute to risk for METH abuse and progression to addiction among humans.

There is now a substantial literature examining choice of psychostimulants over competing reinforcers (Kerstetter et al. 2012; Perry et al. 2013; Nader et al. 2014; Caprioli et al. 2015a, 2017; Venniro et al. 2017, 2021, 2022; Sedighim et al. 2021; Vandaele and Ahmed 2022). Cocaine choice studies have established that there are considerable interindividual differences in cocaine choosing behavior, in addition to overarching effects of subject sex and interval between reinforcer availability on cocaine preference (Kerstetter and Kippin 2011; Kerstetter et al. 2012; Perry et al. 2013; Vandaele et al. 2016; Vandaele and Ahmed 2022). Conversely, in the growing literature examining METH choice, selection of METH over either food or social reinforcement is reportedly negligible or absent (Caprioli et al. 2015a, 2017; Venniro et al. 2017, 2022). Such findings are at odds with the high abuse potential of METH and clinical presentation of METH use disorder in humans, suggesting there is a need to refine METH choice models to determine the conditions that promote METH taking at the cost of alternative, natural reinforcers.

The temporal interval between discrete choices has been shown to influence drug preference, where short (e.g., 20 s) intervals increase cocaine selection over food in male and female rats, and long (e.g., 600 s) intervals reduce cocaine selection in favor of alternative reinforcers (Kerstetter et al. 2012; Vandaele et al. 2016; Vandaele and Ahmed 2022). In fact, due to this drug taking suppression effect, reinforcement schedules with long intervals have been used to model “voluntary drug abstinence” (Fredriksson et al. 2021). Notably, the vast majority of METH choice studies have employed a long 600 s interval (Caprioli et al. 2017; Venniro et al. 2017, 2019; Fredriksson et al. 2021). As the impact of short intervals on METH choice has not been thoroughly explored, we compared METH versus food choice with short and long intertrial intervals (ITIs), and across multiple METH doses, in male and female rats. We furthermore included response breakpoint under a progressive ratio (PR) schedule as an index of motivation for METH and food reinforcers in the absence of choice, to investigate the relatedness of METH choice to this more well-established measure of an addiction-related rat behavior.

## Methods and materials

### Subjects

Male and female Sprague-Dawley rats (Charles River, Hollister, CA) were pair-housed, in a temperature- and humidity-controlled vivarium on a 12 h light-dark cycle. In Experiment 1, food (rat chow, Harlan, Indianapolis, IN) access was initially restricted to 20 g for females and 25 g for males per day, then to 40 g per day for females and males. In a separate study, food access in the home cage was found to have no influence on operant behavior. Thus, food access was not restricted in Experiment 2. All rats had *ad libitum* access to water. The housing and care of the rats followed the *Guidelines for the Care and Use of Mammals in Neuroscience and Behavioral Research* (National Research Council 2003) and was approved by the Institutional Animal Care and Use Committee at the University of California Santa Barbara.

### Intravenous catheter surgery

Male and female rats were anesthetized via isoflurane gas (5% for induction; 2.5% for maintenance), and a jugular catheter was implanted, as described previously (Kerstetter et al. 2008). Chronic indwelling catheters were constructed using a bent steel cannula with a screw-type connector (Plastics One, Roanoke, VA), silastic tubing (10 cm, i.d. 0.64 mm, o.d. 1.19 mm; Dow Corning, Midland, MI), polypropylene mesh (Atrium Medical, Hudson, NH), and cranioplastic cement. The catheter was inserted into the right jugular vein, secured to surrounding tissue, and run subcutaneously to exit posterior to the shoulder blades. All rats were allowed a minimum of five days to recover from surgery prior to beginning operant training.

### Drug treatments and solutions

Catheters were flushed prior to operant sessions with ticarcillin disodium/clavulanate potassium (Timentin; 10 mg in 0.1 ml; Schein Pharmaceutical, Florham Park, NJ) dissolved in 0.9% physiological saline (Experiment 1), or with cefazolin (10 mg in 0.1 ml; Covetrus North America, Visalia, CA) dissolved in sterile water and heparin (70 U) (Experiment 2), and with 0.1 ml of heparinized saline (6.0 U in 0.1 ml 0.9% physiological saline) after operant sessions as a prophylactic measure against microbial infection and to extend catheter patency. Catheter patency was verified by infusing 0.12 ml (males) or 0.04 ml (females) of brevital sodium (10 mg/ml i.v.; Eli Lilly, Indianapolis, IN), which produces a rapid loss of muscle tone only when administered intravenously.

Methamphetamine HCl (Sigma, St. Louis, MO) was dissolved in sterile saline, and solutions were filtered using a 0.45 mm, 0.22 μm ultracleaning filter unit (Fisher Scientific, Los Angeles, CA). Solutions were prepared at different concentrations to deliver different doses (0.05, 0.1, or 0.2 mg/kg) in 0.1 ml intravenous (IV) infusions.

### Apparatus

Rats were trained in operant chambers located inside sound-attenuating cubicles, fitted with an electric fan and controlled by a Med Associates system. The operant chambers were equipped with two retractable levers, a stimulus light above each lever, a food pellet dispenser between the levers, and an infusion line connected to a pump (Med Associates, Fairfax, VT). The external ports of the implanted catheters were connected to a liquid swivel (Instech, Plymouth Meeting, PA) via polyethylene 20 tubing encased in a steel spring leash (Plastics One, Roanoke, VA); the swivel was suspended above the operant conditioning chamber and connected via polyethylene tubing to an infusion pump (Med Associates, Fairfax, VT).

### Self-administration procedures

#### Acquisition of operant responding

Rats were trained to lever press on the right lever for food and on the left lever for METH on alternating days for a minimum of six days and a maximum of twelve days, until they reached at least 20 (out of 25 possible) reinforcers per session for two consecutive sessions for both reinforcers. For training sessions, only one lever was extended (i.e., only METH or food available), and rats were trained to press the lever for reinforcement under a FR1 schedule of reinforcement with either a 20 or 600 s ITI. Training sessions were balanced to ensure that rats had equal experience in responding for food and METH reinforcement prior to choice sessions. Operant sessions lasted 6 h or until the rat earned a total of 25 reinforcers. A maximum limit of 25 reinforcers per session was imposed to minimize reinforcement history differences across subjects and reinforcers, as well as to ensure the health and safety of the rats, specifically when administering METH under higher dose and short ITI conditions.

During food training sessions, responses on the right lever resulted in the delivery of two (for females) or three (for males) grain food or banana-flavored sucrose pellets (45 mg) into the food pellet dispenser and the illumination of a white stimulus light above the food lever for five seconds. These differences in food reinforcement (two or three pellets) approximate differences in body weight and were previously shown to produce equivalent operant responding in males and females (Kerstetter et al. 2008). After food pellet delivery and illumination of the stimulus light above the food-paired lever, there was a 20 or 600 s ITI during which the lever was retracted.

During METH training sessions, responses on the left lever resulted in the delivery of a METH (IV) infusion via activation of the infusion pump for four seconds, and the illumination of a white stimulus light above the METH lever for five seconds. After the infusion and illumination of the stimulus light above the METH-paired lever, there was a 20 or 600 s ITI during which the lever was retracted.

In Experiment 1, high-fat food pellets were used as the food reinforcer; rats were allowed to self-administer METH at multiple doses (0.05, 0.1, and 0.2 mg/kg/infusion, IV; within-subjects design), and a 20 s ITI was employed, to compare the dose-response for METH reinforcement in males and females. In Experiment 2, banana-flavored sucrose pellets were used as the food reinforcer to confirm findings were replicable with different types of food reward; rats self-administered METH (0.05 or 0.1 mg/kg/infusion, IV; between-subjects design), and the 20 s and 600 s ITIs were employed, to examine the impact of ITI on METH reinforcement.

#### METH versus food choice schedules

Choice sessions were conducted in a manner similar to what was described previously for cocaine choice (Kerstetter et al. 2012) and allowed selection of METH or food reinforcers using the same operant parameters as the single reinforcer FR1 training sessions that preceded the choice sessions (i.e., choice sessions employed the same number and type of food pellets, dose of METH, illumination of the conditioned light stimulus, and ITI that were employed in the training sessions immediately prior). During choice sessions, both levers were extended, and rats were able to choose freely between METH and food reinforcement. Each response resulted in delivery of the selected reinforcer and illumination of the respective lever-paired stimulus light, followed by an ITI, during which the levers were retracted and no further reinforcement was available; the next discrete trial was signaled by the extension of the levers. All rats underwent five to six choice sessions with each session having a maximum of 25 discrete trials; sessions lasted up to 6 h or until all 25 trials were completed. The same doses and ITIs were employed in Experiments 1 and 2 during choice sessions as were used in training sessions. In Experiment 2, the 600 s ITI was included as a comparison to match procedures employed in other laboratories (Lenoir et al. 2007; Lenoir & Ahmed 2008; Caprioli 2015).

#### Progressive ratio (PR) schedules

With the exception of schedule, all PR tests used the same reinforcement parameters (i.e., number of food pellets, METH doses, and lever associated with delivery of each reinforcer) as were used during the acquisition and discrete choice sessions. During the PR sessions, the number of responses needed for food pellet or drug infusion delivery within each session were progressively increased according to the following sequence: 1, 2, 4, 9, 12, 15, 20, 25, 32, 40, 50, 62, 77, 95, 118, 145, 178, 219, 268, 328, 402, 492, 603, etc. (Richardson and Roberts 1996). PR sessions lasted until a 30-min period elapsed between reinforcer delivery. Food and METH sessions alternated across six daily sessions.

### Statistical analysis

Data were analyzed using GraphPad (Prism 10, La Jolla, CA, USA). For choice session data, percent METH choice was calculated by dividing the number of METH infusions obtained by the total number of reinforcers obtained and multiplying by 100 (i.e., [infusions/infusions + pellets]*100); METH choice values were averaged across the last four choice sessions completed. Reinforcer (METH versus food) preferences were determined using 60 and 40% METH choice cutoffs, respectively, and intermediary choice scores were categorized as preferring neither reinforcer. For PR schedules, the final completed response ratio (i.e., breakpoint, as defined in Richardson and Roberts 1996) was averaged across three sessions for each reinforcer. In Experiment 1, data were analyzed using ANOVAs with repeated measures for within-subject factors (reinforcer and dose). In Experiment 2, data were analyzed using two- and three-way ANOVAs. Significant main and interaction effects (*p* < 0.05) were followed up with Bonferroni post hoc tests for pairwise comparisons. Reinforcer preference proportions were compared across groups using the Chi-square test or the Fisher’s exact test where appropriate. The relationship between METH choice and the PR breakpoint ratio (METH/food) was assessed using the Pearson correlation coefficient. Outliers were identified using 2.5 standard deviations from the mean as a cutoff. Results were considered statistically significant when *p* < 0.05.

## Results

### Experiment 1: impact of sex and dose on METH versus food choice using short (20 s) ITI

#### METH choice

**Fig. 1:**
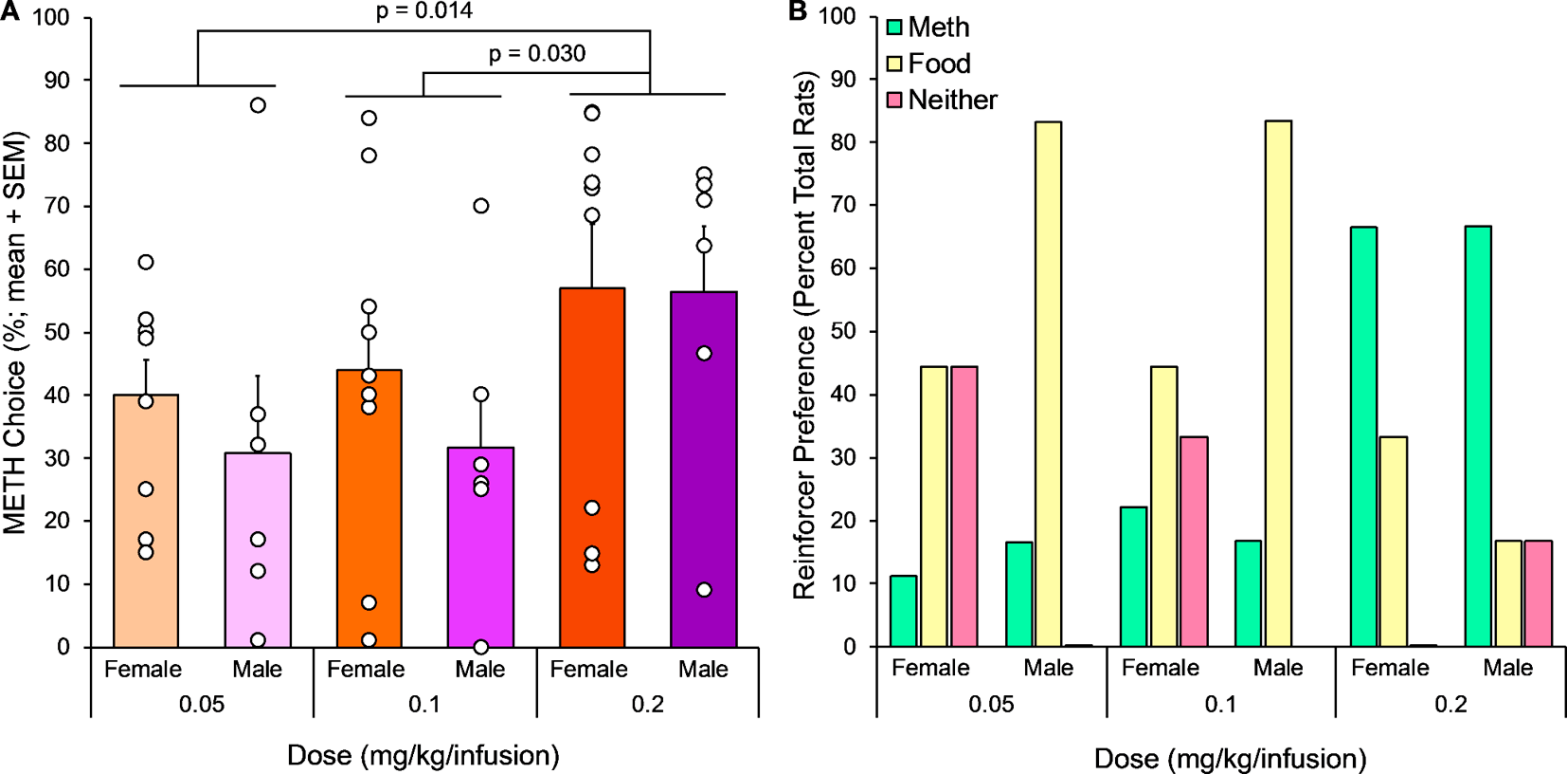
Experiment 1, METH versus food discrete choice (*n* = 6–9). (**A**) Percent METH choice dose response in female and male rats. Percent METH (versus food) choice across discrete choice sessions with 20 s ITI was affected by dose but not sex. Mixed ANOVA, main effect of dose, *p* = 0.010. Bonferroni post hoc tests shown on graph. (**B**) Number of rats preferring METH infusions, sucrose food pellets, or neither reinforcer, using 60 and 40% METH preference as preference group cutoffs. The proportion of rats preferring METH versus food versus neither differed significantly between sex-dose groups. Fisher’s exact test, *p* = 0.029

Rats learned to respond on separate levers for METH (0.05, 0.1, or 0.2 mg/kg/infusion) or food pellets and were then tested for choice between reinforcers with short ITIs. Percent METH choice was significantly impacted by METH dose but not subject sex (Fig. 1A). There was a significant main effect of dose on METH choice (dose, F(2,26) = 0.5.59, *p* = 0.010) with METH choice being significantly higher among rats receiving the highest dose, compared to the two lower doses (0.2 versus 0.05 mg/kg/infusion, *p* = 0.014; 0.2 versus 0.1 mg/kg/infusion, *p* = 0.030). In contrast, there was not a significant effect of sex or a significant interaction between sex and dose. We also examined the relative proportion of rats preferring METH versus food or showing no preference between reinforcers. We found that reinforcer preferences were unevenly distributed across all subgroups (Fisher’s exact, *p* = 0.029; Fig. 1B).

#### Breakpoint

**Fig. 2:**
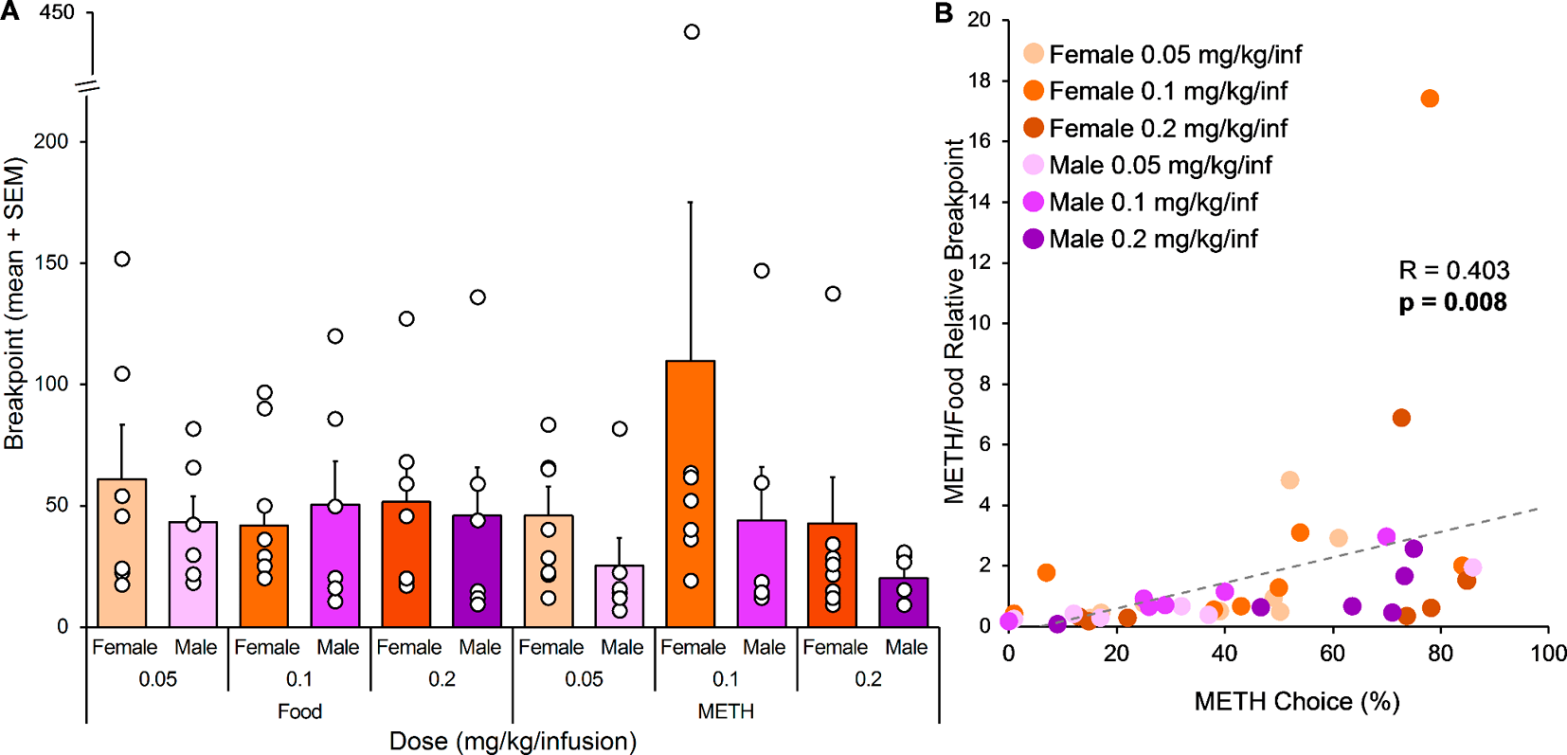
Experiment 1, Progressive Ratio (*n* = 6–8). (**A**) PR breakpoints for food and METH reinforcers across METH doses in female and male rats. Breakpoints did not differ between food and METH reinforcers and were not influenced by dose or sex (assessed with mixed ANOVAs). The high METH breakpoint value in the 0.1 mg/kg/infusion female group is not an outlier, using 2.5 standard deviations above the mean as a cutoff. (**B**) Correlation between percent METH choice and the ratio between METH and food PR breakpoints. Percent METH choice was significantly positively correlated with the ratio between METH and food breakpoints. Pearson’s R and p-value shown on graph

Progressive ratio breakpoints were compared across METH doses in female and male rats for METH and food reinforcers (Fig. 2A). There was no main effect of sex or dose, and no significant interaction between sex and dose, on METH or food breakpoints.

#### Correlations

The ratio of METH breakpoint to food breakpoint (hereafter, relative breakpoint) was calculated to serve as an index of relative motivation for drug versus food reinforcers. The relationship between METH choice and relative breakpoint was assessed across all subjects (Fig. 2B). METH choice was significantly positively correlated with the relative breakpoint (*R* = 0.403, *p* = 0.008), indicating that an increase in METH choice was associated with an increase in relative motivation for the METH versus food reinforcer.

### Experiment 2: impact of ITI on METH versus food choice

#### METH choice

**Fig. 3:**
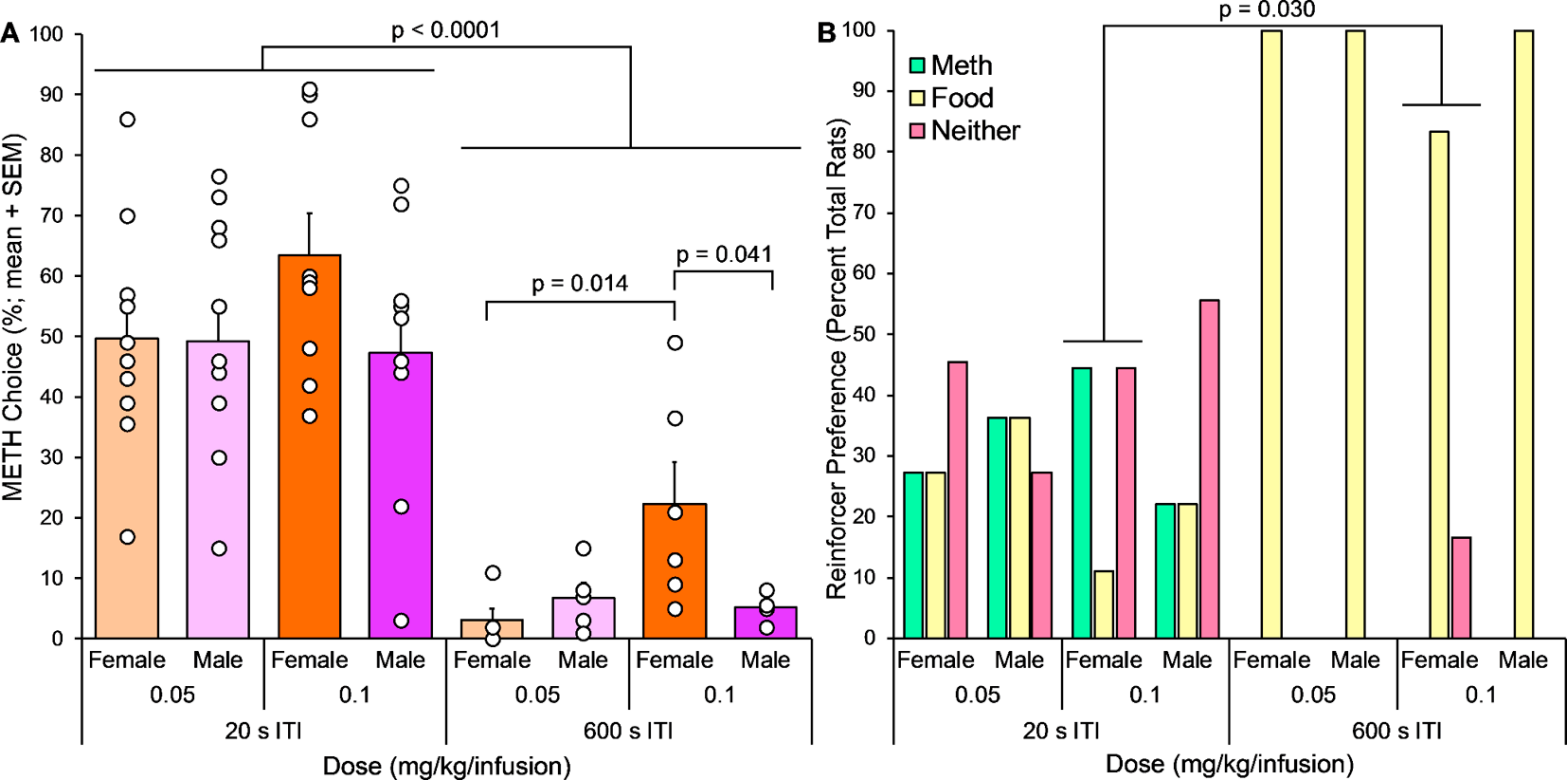
Experiment 2, METH versus food discrete choice (*n* = 4–11). (**A**) Percent METH choice comparison between 20 s and 600 s ITI across two METH doses and in female and male rats. Percent METH (versus food) choice across discrete choice sessions differed significantly with 20 s versus 10 min ITI. Three-way ANOVA, main effect of ITI, *p* < 0.0001. Percent METH choice within sessions using 20 s ITI was not affected by dose or sex; percent METH choice with 600 s ITI was affected by an interaction between sex and dose. Two-way ANOVA, sex-dose interaction, *p* = 0.039. Bonferroni post hoc tests shown on graph. (**B**) Number of rats preferring METH infusions, sucrose food pellets, or neither reinforcer, using 60 and 40% METH preference as preference group cutoffs. The proportion of rats preferring METH versus food versus neither reinforcer differed significantly across all sex-dose-ITI groups (*p* = 0.007). Preference proportions differed between 20 s and 600 s ITI conditions among female rats receiving the higher METH dose (Fisher’s exact test, *p* = 0.030)

Following training (as above), the percent METH choice was examined when trials were separated by either short (20 s) or long (600 s) intervals across two METH doses in male and female rats (Fig. 3A). There was a significant effect of ITI on METH choice (ITI, F(1,52) = 76.5, *p* < 0.0001). The effect of ITI was consistent across both doses and sexes; with the short 20 s ITI, there was higher METH choice than with the long 600 s ITI in male rats receiving 0.05 mg/kg/infusion METH (20 s versus 600 s ITI, *p* < 0.001) and 0.1 mg/kg/infusion METH (20 s versus 600 s ITI, *p* = 0.003). The short ITI also increased METH choice compared to the long ITI in female rats receiving both doses of METH (20 s versus 600 s ITI, both *p* < 0.001).

Percent METH choice was subsequently analyzed separately for the 20 s and 600 s ITIs to examine dose and sex effects within each ITI schedule condition. While there was no impact of sex or dose on METH choice at the short 20 s ITI, there was a significant interaction between sex and dose on METH choice with the long 600 s ITI (sex x dose, F(1,16) = 5.03, *p* = 0.039). With the 600 s ITI, female rats receiving 0.1 mg/kg/infusion METH exhibited higher METH choice compared to females receiving the lower 0.05 mg/kg/infusion METH dose (*p* = 0.014), and also exhibited higher METH choice compared to males receiving the same 0.1 mg/kg/infusion METH dose (*p* = 0.041).

The proportion of rats preferring METH versus food versus neither reinforcer varied significantly in distribution across all sex-dose-ITI subgroups (X^2^(14) = 30.2, *p* = 0.007; Fig. 3B). Reinforcer preference proportions also varied significantly across sex-ITI groups among rats receiving the 0.05 mg/kg/infusion (Fisher’s exact, *p* = 0.041) and the 0.1 mg/kg/infusion (Fisher’s exact, *p* = 0.014) METH doses, and across dose-ITI groups among female (Fisher’s exact, *p* = 0.013) and male (Fisher’s exact, *p* = 0.035) rats, but not across sex-dose groups among 20 or 600 s ITIs. Reinforcer preference proportions differ significantly between the 20 s and 600 s ITIs among the female rats receiving the 0.1 mg/kg/infusion METH dose.

#### Breakpoint

**Fig. 4:**
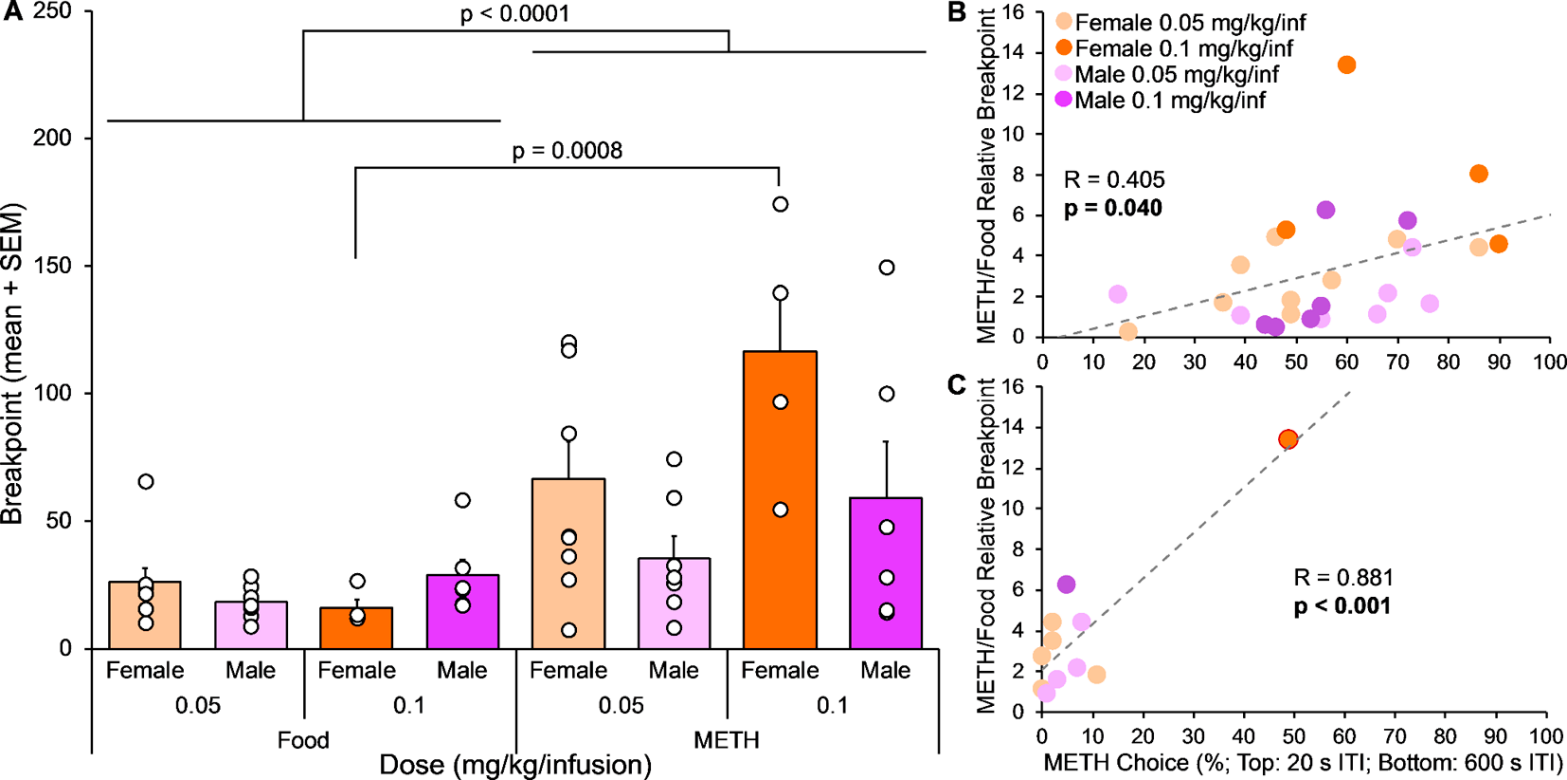
Experiment 2, Progressive Ratio (*n* = 4–9). (**A**) PR breakpoints for food and METH reinforcers across two METH doses in female and male rats. Breakpoints were significantly different for METH versus food reinforcers and were affected by sex and an interaction between sex and reinforcer. Three-way ANOVA, main effect of reinforcer, *p* < 0.0001; main effect of sex, *p* = 0.029; reinforcer-sex interaction, *p* = 0.016. Bonferroni post hoc test shown on graph. (**B**) Correlation between percent METH choice with 20 s ITI and the METH to food breakpoint ratio. METH choice across sessions with 20 s ITI was significantly positively correlated with the ratio between METH and food breakpoints. Pearson’s R and p-value shown on graph. (**C**) Correlation between percent METH choice with 600 s ITI and the METH to food breakpoint ratio. METH choice across sessions with 600 s ITI was significantly positively correlated with the ratio between METH and food breakpoints; however this was driven entirely by a single female rat in the 0.1 mg/kg/infusion dose group. Pearson’s R and p-value shown on graph

Progressive ratio breakpoints were compared across two METH doses in female and male rats for METH and food reinforcers (Fig. 4A). Breakpoints differed significantly between METH and food rewards (reinforcer F(1,44) = 25.6, *p* < 0.001). This was most evident among female rats receiving the highest METH dose (METH versus food, *p* < 0.001). There was also a significant effect of sex on breakpoints in general (sex, F(1,44) = 5.08, *p* = 0.029), and there was a significant interaction between reinforcer and sex (reinforcer x sex, F(1,44) = 6.31, *p* = 0.016). Breakpoints were subsequently analyzed separately for METH and food reinforcers. While there was no significant effect of sex or dose on breakpoints for food, there was a significant effect of sex on the breakpoints for METH (sex, F(1,22) = 6.17, *p* = 0.021), as well as a trending effect of dose (dose, F(1,22) = 4.25, *p* = 0.051). Although there were no significant post hoc pairwise comparisons, breakpoints for METH were nominally higher among females compared to males within dose, and at the higher versus lower METH dose within sex.

#### Correlations

The ratio of METH breakpoint to food breakpoint was again calculated to serve as an index of relative motivation for drug versus food reinforcers, and the relationships between METH choice (20 s and 600 s ITI) and the relative reinforcer breakpoint were assessed across all subjects (Fig. 4B). METH choice with the 20 s ITI was significantly positively correlated with the METH/food relative breakpoints (*R* = 0.405, *p* = 0.040), consistent with an increase in METH choice being associated with an increase in relative motivation for the METH versus food reinforcer. Similarly, when the 600 s ITI was employed, METH choice was significantly positively correlated with the METH/food relative breakpoint (*R* = 0.881, *p* < 0.001); however, the significance of this correlation is driven entirely by a single female rat in the higher dose group.

## Discussion

The present study demonstrates that rats exhibit heterogenous propensities for selecting METH over food reinforcement in a manner that is strongly modulated by reinforcement schedule. Under the short (20 s) ITI, we found that METH choice was dose dependent but varied widely, with male and female rats exhibiting a combination of METH, food, and no reinforcer preference. In sharp contrast, the long (600 s) ITI markedly suppressed METH choice, eliminating preference for the drug entirely, regardless of dose, and among both female and male rats. Although the female rats chose the higher METH dose more frequently than the lower dose, and more frequently than the males chose either METH dose, the females still chose food over METH in a majority of discrete choice trials. In fact, when using the 600 s ITI, all rats exhibited preference for the food reinforcer, choosing sucrose pellets in more than 55% of trials, except for one female rat who had no preference between food and the higher METH dose.

Consistent with studies employing other drugs of abuse, choice of METH over a competing reinforcer is critically dependent upon the interval between opportunities to choose. In rodent studies, METH preference over alternatives, including sucrose or social interaction, is considered an extremely rare phenotype. However, prior METH choice studies employed a long (480–600 s) ITI, resulting in METH selection in fewer than 15% of discrete trials, and although a small subgroup tends to choose METH more frequently, a METH preferring phenotype was not observed in any individual rat (Caprioli et al. 2017; Venniro et al. 2017, 2018; Rossi et al. 2020; Venniro and Shaham 2020). Similarly, under long (480–600 s) ITIs, heroin was chosen in very few trials (i.e., less than 15%), and no rats preferred heroin, whereas cocaine was selected in approximately 25% of trials, and a small minority (around 10%) of rats exhibited an overall preference for cocaine over non-drug reinforcers (Cantin et al. 2010; Vandaele et al. 2016, 2019; Venniro et al. 2017, 2018, 2021; Freese et al. 2018; Venniro and Shaham 2020).

Critically, we found that using a shorter ITI dramatically shifted METH choice behavior, with METH infusions being chosen over food pellets more frequently in a dose-dependent manner, compared to under long ITI. Similarly, when shorter ITIs are employed, choice of other drugs over non-drug alternatives increases (Kerstetter and Kippin 2011; Kerstetter et al. 2012; Vandaele et al. 2016; Russo et al. 2018; Townsend et al. 2021). For example, in a study examining saccharin versus alcohol choice, rats obtained more alcohol and less saccharin when a 120 s ITI was used, compared to a 300 s ITI (Russo et al. 2018). When the effect of short (20–60 s) and long (600 s) ITIs are compared directly, choice of cocaine infusions shifts from approximately 75–80% to 25–35% (Vandaele et al. 2016). This association between short ITI and high drug choice appears to generalize broadly to drugs of abuse; in one study using a 20 s ITI alone, choice of drug infusions over a food reinforcer was highly dose-dependent, with the highest drug dose chosen in over 80% of discrete choice trials for METH, amphetamine, cocaine, heroin, and fentanyl (Townsend et al. 2021). However, METH choice had never been directly compared using different ITIs; thus, our findings confirm for the first time that decreasing the interval between discrete choice trials in turn increases METH choice over a food reinforcer, as occurs with cocaine and alcohol.

Vandaele et al. (2016); (2022) posit that the use of short ITIs (i.e., less than 600 s) results in the choice between cocaine and saccharin occurring while the rat is “under the influence” of the drug and still experiencing acute behavioral and physiological drug effects, which in turn promotes a bias towards choosing the drug or, conversely, suppresses selection of the alternative reinforcer. Consistent with this hypothesis, choosing while not acutely intoxicated (i.e., using a 600 s ITI), most rats preferred saccharin to cocaine infusions, but exposure to a priming dose of cocaine immediately prior to choice sessions dose-dependently shifted preference towards the drug, demonstrating that acute intoxication promoted selection of the drug over the food reinforcer (Vandaele et al. 2016; Freese et al. 2018). Our findings also align with this hypothesis; under the short ITI, rats make discrete choices while in a more acutely intoxicated state, leading to enhanced preference for METH over food. The effect of intoxication on drug choice, however, may not arise from differences in pharmacokinetics. Cocaine has a relatively short half-life in rats compared to METH (~ 15 versus 55 min, respectively), such that the decreases in brain drug concentrations and pharmacological effects between a short (e.g., 20 s) and a long (e.g., 600 s) post-infusion interval are expected to be more substantial for cocaine than for METH (Booze et al. 1997; Ma et al. 1999; Riviere et al. 2000). Drug choice decreases under long (versus short) ITIs for both cocaine and METH, but the effect on cocaine choice (as observed in Kerstetter et al. 2012; Vandaele et al. 2016; Vandaele and Ahmed 2022) is smaller than the effect presently observed with METH choice. Indeed, the suppression of METH choice caused by the availability of an alternative reinforcer under a 600 s ITI is so robust that it has frequently been used as a model of voluntary abstinence from METH self-administration (Caprioli et al. 2015b, 2017; Venniro et al. 2017). Therefore, while the “under the influence” effect clearly applies to both cocaine and METH choice, the counter condition (i.e., not under the influence) is not easily explained in terms of diminished drug concentrations in the body.

Since the impact of short versus long ITI on drug choice is consistent across drugs with differing pharmacodynamics and pharmacokinetics, another potential mechanism contributing to this effect could involve an interplay between reinforcement schedule and reward dynamics more broadly. For instance, imposing delays on reinforcer availability could differentially enhance the motivational value of drug versus non-drug reinforcers. FR responding frequency is higher and response latency is shorter, for non-drug versus drug reinforcers, when tested separately, irrespective of the specific non-drug reinforcer or drug tested (Vandaele et al. 2016, 2019; Freese et al. 2018; Russo et al. 2018; Venniro et al. 2021). Similarly, we found that, during acquisition sessions using the 20 s ITI, the mean reinforcement rate for sucrose pellets (~ 0.8 deliveries per min) was more than double the rate of METH infusions obtained (~ 0.3 per min), indicating a tendency to respond for sucrose pellets with higher frequency (Online Resource 1). Meanwhile, under the 600 s ITI, the response rate was limited to a maximum of 0.1 reinforcers/min for each reinforcer (Online Resource 1). Thus, increasing the ITI from 20 to 600 s imposes a greater relative delay on the availability of the non-drug reinforcer versus the drug reinforcer. The greater relative limitation placed on the ability to respond for the non-drug option relative to the drug option may differentially enhance the motivational value of non-drug compared to drug reinforcement, leading to higher non-drug choice under longer ITI schedules. Further direct investigation is needed to validate this hypothetical explanation for the sensitivity of METH (and cocaine) choice to interval duration.

The current study examined PR breakpoints, which are often used as an index of motivation to obtain drugs and other reinforcers, and we assessed the relationship between METH choice and relative breakpoint as a measure of construct validity. Generally, breakpoints were higher for METH than food, and METH breakpoints were higher for females than males, suggesting greater motivation to acquire METH. Across experiments, there was consistently a strong significant positive correlation between METH choice under the 20 s ITI schedule and the relative breakpoint, suggesting a strong association between favorability of METH over food and the relative motivation to acquire METH versus food under a PR schedule. In contrast, although there was a significant correlation between the 600 s ITI METH choice and the relative breakpoint, this relationship was driven entirely by a single female rat; when this data point is removed, the association and statistical significance disappear. This is largely consistent with the literature, in which drug choice under a 600 s ITI schedule is generally discordant with PR responding for drug versus non-drug reinforcers. Depending on drug self-administration training history, METH infusions obtained under a PR schedule were either no different or higher than food rewards acquired, despite very low METH choice and a persistent preference for food (Caprioli et al. 2015a). Concordance with other behavioral measures relevant to addiction is mixed; for example, drug choice under a 600 s ITI was correlated with a different behavioral economic measure of willingness to work for the drug reinforcer (termed, essential value) (Kearns et al. 2017; Schwartz et al. 2017). Yet METH choice (over food or social rewards) also failed to differentiate between low, medium, and high addiction score subgroups, which were defined by differential responding for METH under a PR schedule, in an extinction paradigm, and when drug delivery was punished by a foot shock co-delivery (Venniro et al. 2018). Thus, the 600 s ITI METH choice model may lack some construct validity due to its divergence from other established measures of addiction-like behaviors in rodents. Conversely, the short ITI drug choice model has demonstrated predictive validity for screening pharmacotherapeutics. For example, buprenorphine exposure dose-dependently reduced choice of heroin infusions over a food reinforcer under a 20 s ITI schedule, consistent with the efficacy of buprenorphine as a treatment for opioid use disorder (Townsend et al. 2021). Further comparison of METH choice, employing a short (e.g., 20 s) ITI, with other meaningful outcome measures should be conducted to further validate the utility of the 20 s ITI METH choice model.

Meaningful interrogation of the propensity for some individuals to compulsively use METH in favor of other naturally rewarding activities relies on our ability to examine the physiological, neurobiological, and environmental factors that contribute to relevant behavioral outcomes. Using a short ITI produces a wide range of METH choice profiles, with some individuals exhibiting robust METH preference and other exhibiting robust food preferences. These variable preference profiles may enable detection of factors (e.g., genetics, stress history, etc.) contributing to vulnerability or resilience to drug addiction-like behavior. Moreover, our ability to investigate mechanisms of risk reduction and identify treatments for psychostimulant use disorder requires the ability to statistically evaluate interventions that decrease addiction-like behaviors in rodent models. In contrast to drug choice studies employing the long ITI schedule, METH choice under a short ITI is wider ranging and generally moderate, which notably permits the examination of factors that will reduce METH choice levels and, thus, the screening of putative METH abuse management strategies.

## Electronic supplementary material

Below is the link to the electronic supplementary material.

Supplementary Material 1

## Data Availability

Supporting data and material can be found in the additional files and can be requested from the corresponding author.
