# Supplementary material for: Time to choose: impact of intertrial interval on selecting between methamphetamine and food reinforcement in male and female rats
Source: Psychopharmacology (Berl). 2025 Feb 8;242(4):693–702. doi: 10.1007/s00213-025-06750-w (PMC11890418; doi:10.1007/s00213-025-06750-w)
Supplement: Supplementary file 1 — Supplementary Material 1 [file 213_2025_6750_MOESM1_ESM.docx]

Psychopharmacology

**Time to choose: impact of inter-trial interval on selecting between methamphetamine and food reinforcement in male and female rats**

Marlaina R. Stocco, Mari Purpura, Philip A. Vieira, Kira Wallquist, Sijia Wang, Julia Adams, Karen K. Szumlinski, and Tod E. Kippin

Corresponding Author: Dr. Marlaina R. Stocco

Department of Psychological and Brain Sciences, University of California, Santa Barbara

[marlainastocco@ucsb.edu](mailto:marlainastocco@ucsb.edu)

**Online Resource 1** FR1 Training Reinforcement Rate in Experiment 2. Reinforcer delivery per minute (i.e., reinforcement rate) during acquisition sessions was reinforcer and ITI dependent. Three-way ANOVA, main effect of reinforcer, p = 0.0001; main effect of ITI, p < 0.0001; Reinforcer-ITI interaction, p = 0.0002. Reinforcement rate for food (sucrose pellets per min) was higher under the 20 s ITI than under the 600 s ITI (Bonferroni, p < 0.0001), and was higher for food than for METH (infusions per minute) under the 20 s ITI (Bonferroni, p < 0.0001).
